# Surgical resection of symptomatic brain metastases improves the clinical status and facilitates further treatment

**DOI:** 10.1002/cam4.3402

**Published:** 2020-08-28

**Authors:** Petra Schödel, Stephanie T. Jünger, Maike Wittersheim, Hans Christian Reinhardt, Nils‐Ole Schmidt, Roland Goldbrunner, Martin Proescholdt, Stefan Grau

**Affiliations:** ^1^ Department of Neurosurgery University Medical Center Regensburg Regensburg Germany; ^2^ Faculty of Medicine and University Hospital Center for Neurosurgery Dept of Neurosurgery University of Cologne Cologne Germany; ^3^ Faculty of Medicine and University Hospital Department of Pathology University of Cologne Cologne Germany; ^4^ Faculty of Medicine and University Hospital Clinic I of Internal Medicine University of Cologne Cologne Germany; ^5^ Faculty of Medicine and University Hospital Center for Integrated Oncology University of Cologne Cologne Germany; ^6^ Faculty of Medicine Center for Molecular Medicine University of Cologne Cologne Germany; ^7^ Cologne Excellence Cluster on Cellular Stress Response in Aging‐Associated Diseases (CECAD) University of Cologne Cologne Germany; ^8^ University Medical Center Regensburg Wilhelm Sander Neuro‐Oncology Unit Regensburg Germany

## Abstract

**Background:**

Brain metastases (BM) frequently cause focal neurological deficits leading to a reduced Karnofsky performance score (KPS). Since KPS is routinely used to guide the choice of adjuvant therapy, we hypothesized that improving KPS by surgical resection may improve the chance for adjuvant treatment and ultimately result in better survival. We therefore analyzed the course of a large cohort undergoing resection of symptomatic brain metastases in the context of further treatment and clinical outcome.

**Patients and methods:**

In a bi‐centric retrospective analysis we retrieved baseline, clinical, and treatment‐related parameters of patients operated on BM between 2010 and 2019. Survival was calculated using Kaplan‐Meier estimates; prognostic factors for survival were analyzed by Log‐rank test and Cox proportional hazards.

**Results:**

We included 750 patients with a median age of 61 (19‐87) years. The functional status was significantly improved by surgical resection, with a median preoperative (KPS) of 80 (10‐100) increasing to 90 (0‐100) after surgery (*P* < .0001). Moreover, surgery improved the RTOG recursive partitioning analysis (RPA) class from III to I/II in 82 patients. Postoperative local radiotherapy and systemic treatment were associated with significantly longer survival (*P* < .0001 for each). Systemic treatment was provided significantly more frequently in patients with a fair postoperative clinical status (KPS ≥ 70; *P* < .0001). The postoperative clinical status, postoperative radiotherapy, systemic treatment, controlled systemic disease and < 4 BM were independent predictors for survival.

**Conclusion:**

The resection of symptomatic BM may restore clinical status, so enhancing the likelihood of receiving adjuvant treatment, and therefore leading to improved overall survival.

## Graphical abstract

In patients with symptomatic brain metastases systemic oncological treatment is the strongest factor for further survival. Resection of a symptomatic brain metastasis results in an improved clinical status which facilitates further treatment.

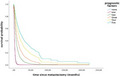

## 1 INTRODUCTION

The number of cancer patients diagnosed with brain metastases (BM) is constantly rising, due to improvement in systemically active anti‐cancer therapies and more sensitive diagnostic techniques.^1^, ^2^ Thus interdisciplinary BM management is constantly changing, particularly in the context of novel systemic treatment options, which are increasingly engineered toward improved blood‐brain‐barrier penetrance.^3^, ^4^, ^5^

Traditionally, the prognosis for patients with BM has been considered extremely poor.^6^, ^7^ In the past, these patients were usually excluded from systemic treatment trials, and tumor‐specific therapy was frequently terminated after BM diagnosis.^8^, ^9^, ^10^ One reason for this was the assumption that central nervous system lesions are rather insensitive to chemotherapy, since the classic therapeutic agents mostly did not pass through the blood‐brain‐barrier to a sufficient extent. However, novel compounds targeting tumor‐specific molecular changes, such as EGFR and BRAF mutations, and ALK fusions, or immunomodulatory agents activating anti‐tumor immune responses, are challenging this paradigm, and some of these drugs show intracerebral efficacy.^3^, ^4^, ^5^, ^8^, ^11^

While surgery has been advocated as a treatment option for BM for almost 30 years,^12^, ^13^ the indication for resection followed by radiotherapy has usually been restricted to singular or solitary, large and/or symptomatic lesions. However, in the light of modern adjuvant radio‐surgical/radio‐therapeutic treatment concepts and the advances in medical treatment mentioned above, the selection criteria for surgery are changing.^1^, ^14^, ^15^, ^16^, ^17^, ^18^

In this context, we investigated the course of patients undergoing surgical treatment for symptomatic brain metastases, focusing on the subsequent use of systemic therapy and its effects on survival.

## 2 MATERIAL AND METHODS

Our data were analyzed in a bi‐centric retrospective study approved by both local ethics committees (University of Cologne approval no. 18‐089; University of Regensburg approval no. 19‐1546‐101).

We queried our institutional databases for adult patients who underwent surgery for BM between 2010 and 2019 and identified demographic and clinical parameters.

The time to diagnosis of BM was calculated from the date of primary tumor diagnosis, until the date of surgery for BM. Post‐surgical survival (PSS) was calculated from the date of BM resection, until death or last follow‐up.

Patients were excluded from the analysis if they had received previous treatment for brain metastases, or if data regarding the postoperative (radio‐)oncological treatment after BM surgery were missing.

The patients´ neurological status was measured using the Medical Research Council‐Neurological Performance Status Scale (MRC‐NPS). Functional performance status was assessed using the Karnofsky performance scale.

Patients were allocated to RTOG recursive partitioning analysis classes.^19^, ^20^

The data originating from patients treated at the University of Cologne were documented using a REDCap database.

Surgery was indicated within an interdisciplinary institutional tumor board, involving board‐certified neurosurgeons, neuro‐oncologists, medical oncologists, neuro‐radiologists, neuropathologists, and palliative care physicians. If required, the procedure was conducted using intraoperative optic navigation and in the case of eloquent location, cortex stimulation awake craniotomy. The extent of resection was assessed by cranial magnetic resonance imaging (cMRI) within 24 to 48 hours postoperatively.

Statistical analysis was performed using SPSS Statistics Version 25 (IBM, Chicago IL). For descriptive statistics, continuous values are given in median and range, ordinal and categorical variables are stated in counts and percentages. Survival rates were estimated using the Kaplan‐Meier method. Univariate analysis (Log‐rank test) was used to identify covariates with an impact on survival after BM resection. Multivariate Cox regression was conducted for significant factors in univariate analysis using the pairwise inclusion method. P‐values lower than 0.05 were considered statistically significant.

## 3 RESULTS

### 3.1 Demographic and baseline clinical data

We identified 805 patients who underwent resection of BM between 2010 and 2019. We excluded 55 patients from the analysis due to missing oncological treatment documentation, leaving 750 patients in the analysis.

The median age at the time of BM diagnosis was 61 years (range 19‐87). Gender distribution was equal, ie 371 (49.5%) male patients. Primary tumors comprised nonsmall cell lung cancer (NSCLC) in 318 (42.4%), melanoma in 114 (15.2%), breast cancer in 116 (15.5%), gastrointestinal tumors in 72 (9.6%), renal cell carcinoma in 24 (3.2%), and others/rare entities in 73 (9.7%) patients; a CUP‐syndrome was diagnosed in 3 (4.4%) patients (Table 1).

**Table 1:** Demographics and baseline characteristics

Parameter	Value
**Age [median; (range)]**	61 (19‐87)
**Male gender [n; (%)]**	371 (49.5)
Primary [n; (%)]	
*Lung*	318 (42.4)
*Melanoma*	114 (15.2)
*Breast*	116 (15.5)
*Gastrointestinal*	72 (9.6)
*Kidney*	24 (3.2)
*CUP*	33 (4.4)
*Others*	73 (9.7)
**Controlled primary disease [n;(%)]**	281 (37.5)
**Number of brain metastases (range)**	1‐34
*Singular/solitary [*n*; (%)]*	462 (61.6)
*Oligo (2‐3) [%]*	185 (24.7)
*Multiple [%]*	103 (13.7)
**Tumor location [n;(%)]**	
*Frontal*	208 (27.7)
*Temporal*	84 (11.2)
*Parietal*	81 (10.8)
*Occipital*	66 (8.8)
*Cerebellar*	196 (26.1)
*More than 1 lobe*	115 (15.3)
Neurological deficits [%]	
*Seizures*	13.5
*Aphasia*	11.3
*Hemiparesis*	21.2
*Visual field defects*	9.3
*Cerebellar signs*	23.2
*Signs of elevated intracranial pressure*	32.7
Postoperative Treatment [n;(%)]	
*Radiotherapy*	613 (81.7)
*Systemic medical treatment*	398 (53.1)
*Modality (Cologne cohort; %))*	
*Chemotherapy*	42.9
*Molecular treatment*	57.1

BM were diagnosed after a median of 55 (0‐312) months after initial tumor diagnosis. BM were synchronous (≤ 3 months of initial diagnosis of cancer) in 316 (42.1%) patients. The systemic status at the time of BM diagnosis was stable in 281 (37.5%) patients.

The number of BM ranged from 1‐34 individual lesions. Grouping resulted in 462 (61.6%) patients with singular or solitary brain lesions, 185 (24.7%) patients with oligo‐metastases (2‐3) and 103 (13.7%) with multiple (≥4) BM. Tumor location and neurological symptoms at diagnosis of BM are listed in Table 1.

**Figure 1:**
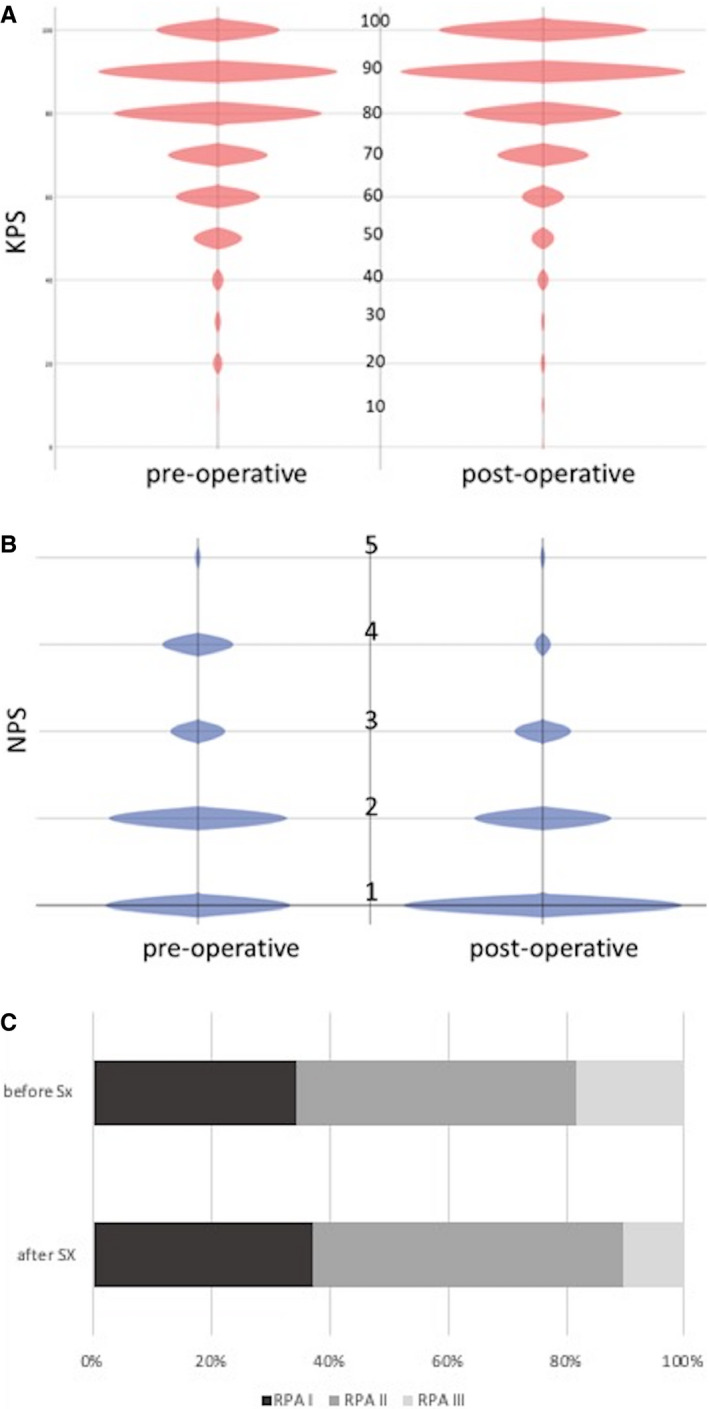
(A‐C), Descriptive account of clinical parameters before and after resection of BM. (A) distribution of KPS, (B) classification of neurological symptom burden according to NPS (C) allocation of patients to RPA classes pre‐ and postoperatively

The median KPS was 80 (range 10‐100) prior to surgery. Preoperative neurological status was assessed by the Medical Research Council Neurological Performance Status Scale (MRC‐NPS) (Table 1 and Figure 1B).

### 3.2 Treatment‐related parameters

Complete resection of brain metastases was achieved in 643 (85.7%) patients.

Complications related to surgery occurred in 84 (11.2%) cases, 42 of these were of minor grade. The complications comprised postoperative hemorrhage (n = 22), cardio‐pulmonary embolism (n = 18), cerebrospinal fluid fistula (n = 7), stroke (n = 5), symptomatic pneumatocephalus (n = 1), and wound infection (n = 38).

Sixty‐six (8.8%) patients did not survive the early postoperative phase of 30 days.

Postoperative cerebral radiotherapy was applied in 613 (81.7%) patients. Due to death before start of treatment, poor clinical status, or refusal of treatment, 137 (18.3%) patients did not receive postoperative local treatment. The radio‐oncological treatment modality was heterogeneous due to the different number of metastases and a change of paradigm during the last decades.

Systemic treatment was continued or initiated in 398 (53.1%) patients. This comprised chemotherapy, molecularly targeted treatments, and a combination of both (Table 2).

**Table 2:** Clinical scores and prognostic group allocation

**SCALE**	Before surgery	After surgery	*P*‐value
**Medical Research Council‐Neurological Performance Status Scale (MRC‐NPS) [N; (%)]**			.0001
**1**	261 (34.8)	433 (57.7)	
**2**	289 (38.5)	191 (25.5)	
**3**	110 (14.7)	90 (12.0)	
**4**	77 (10.3)	25 (3.3)	
**5**	13 (1.7)	11 (1.5)	
**Karnofsky Performance Score [Median; Range]**	80 (10‐100)	90 (0‐100)	<.0001
**Rtog Recursive Partitioning Analysis Groups [N;(%)]**			<.0001
**1**	139 (18.5)	145 (19.3)	
**2**	472 (62.9)	526 (70.1)	
**3**	139 (18.5)	79 (10.5)	

The induction of systemic treatment depended on the primary tumor (Melanoma, NSCLC, breast cancer vs others; *P* = .0001), the postoperative clinical status (KPS; *P* < .0001), the systemic disease status (*P* < .0001) and the time since initial diagnosis (synchronous vs metachronous, *P* < .0001).

### 3.3 Clinical and oncological outcome

Before surgery, the median Karnofsky performance status was 80 (10‐100), whereas the postoperative KPS was 90 (range: 10‐100) (*P* < .0001, Wilcoxon) (Figure 1A). Similarly, surgery changed the neurological symptom burden from 550 (73.3%) patients showing a neurological performance score of one or two (ie no neurological deficit or some neurological deficit but function adequate for useful work, using limbs, gross speech, or few/no visual disturbances) preoperatively, to 624 (83.2%) after surgery (*P* = .0001, Wilcoxon) (Figure 1B).

Preoperative classification of these patients according to Radiation Therapy Oncology Group (RTOG) recursive partitioning analysis (RPA) classes resulted in 139 (18.5%) patients in class I, 472 (62.9%) in class II and 139 (18.5%) in class III. Surgery changed this allocation into 145 (19.3%) in class I, 526 (70.1%) in class II, and 79 (10.5%) in class III (*P* < .0001; Wilcoxon) (Figure 1C).

**Figure 2:**
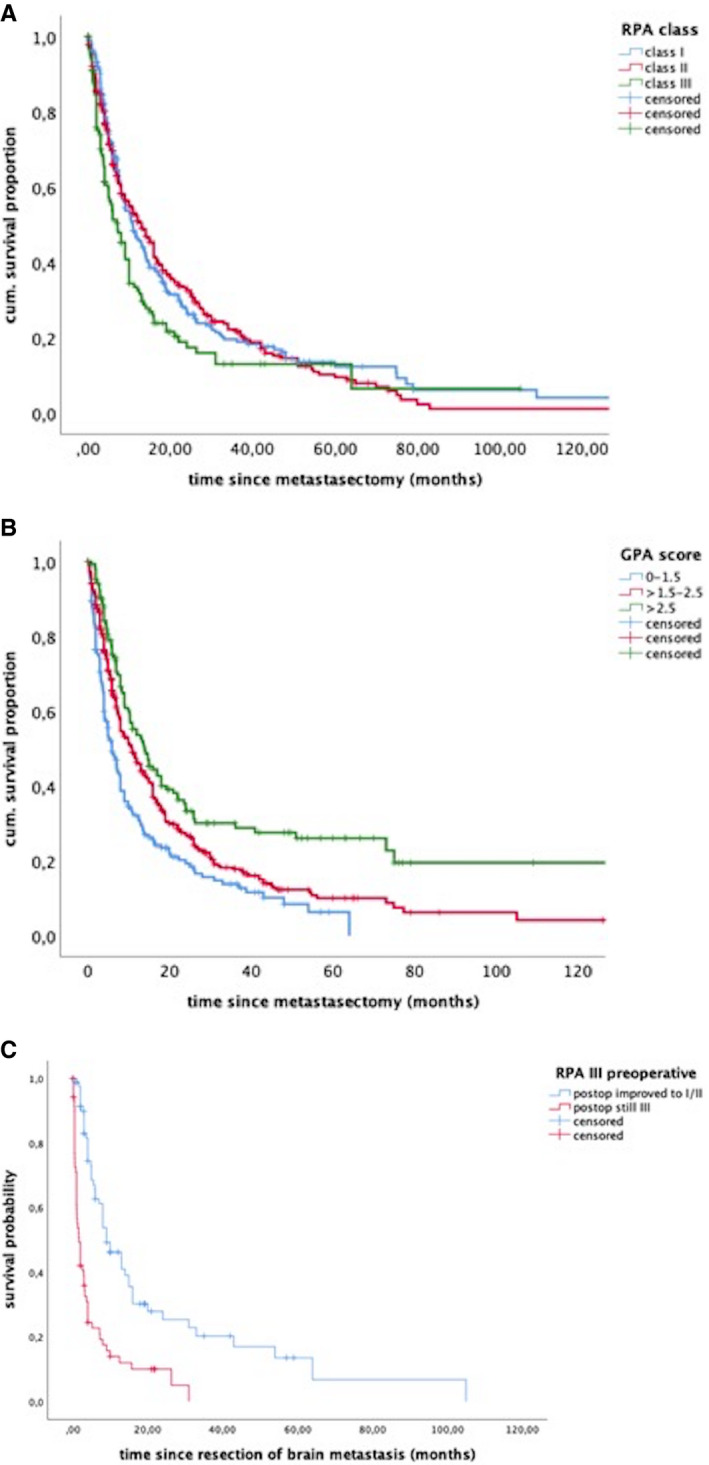
Survival estimates of the patient cohort after allocation to (A) RPA classes, (B) GPA scoring groups and (C) for patients with a preoperative RPA class III stratified according to the postoperative allocations into better classes or still class III

The RPA classification as well as the GPA scoring pre‐ and postoperatively predicted different survival rates (*P* < .0001) (Figure 2A & B). Patients who were allocated to class III prior to surgery (n = 139) but improved in clinical status and could be allocated to class I or II after surgery (n = 82), showed survival estimates corresponding to the new, postoperative class (Figure 2C). Correspondingly, patients whose clinical status worsened from class I/II before surgery into class III (n = 24), showed significantly reduced survival (*P* < .001).

By the time of analysis, 532 (70.9%) patients had died; the cause of death could be reliably identified in 240 patients and comprised systemic tumor progression in 160 (66.7%) patients, fatal cerebral progression in 64 (26.7%), and nontumor related causes in 16 (6.6%) patients.

Median overall survival after resection of BM was 10.9 months (95% CI 9.5‐12.5).

In univariate analysis, a fair clinical status (KPS ≥ 70) pre‐ and postoperatively (*P* < .0001), single or oligo‐metastatic disease (*P* < .0001) and age ≤ 65 years (*P* = .01) were prognostic factors for survival. Patients with postoperative complications displayed significantly shorter survival (*P* < .001) than those without complications. Systemic disease control significantly influenced survival (*P* = .001), while the extent of resection influenced survival only in patients with singular or solitary metastases (*P* = .007), but was not relevant in patients with more than one BM (*P* = .44), (Table 3).

**Table 3:** Analysis of prognostic factors in uni‐ and multivariate analysis

Parameter	Univariate (log rank) [*P*‐value]	Multivariate (Cox regression) [HR 95%CI; *P*‐value]
*Age ≤ 65*	.01	n.s.
Primary tumor	.167	
Controlled systemic status	.001	0.67 0.55‐0.82 <.0001
Timing (synchronous vs metachronous)	.235	
KPS ≥ 70 preoperative	.001	0.53 0.38‐0.71 <.0001
KPS ≥ 70 postoperative	<.0001	
Surgical complications	.015	n.s.
BM count		
single vs oligo	.348	
single vs multiple	.009	
oligo vs multiple	.081	
single/oligo vs multiple	.001	0.63 0.50‐0.80 <.0001
Postoperative radio‐therapy	.001	0.65 0.52‐0.82 <.0001
Systemic treatment after BM resection	<.0001	0.55 0.45‐0.68 <.0001
Extent of resection	.184	

Continuation or initiation of systemic treatment following BM therapy prolonged median survival from 7.0 months (95% CI: 6.1‐7.9) to 15.9 months (95% CI: 13.6‐18.3; *P* < .0001) (Table 3). The administration of such treatment was significantly associated with a good postoperative clinical status (two tailed Fischer's exact test *P* = .001).

**Figure 3:**
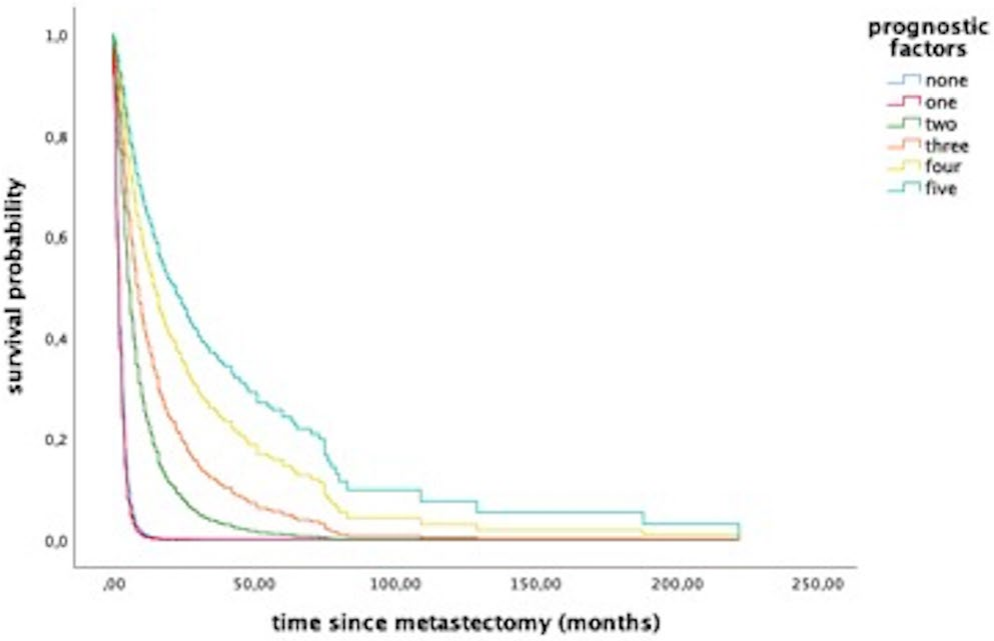
Survival curves demonstrating the impact of prognostic factors from Cox proportional hazards model. These factors were KPS > 70, BM count <4, controlled systemic disease status and postoperative radio‐ and systemic therapy. Curves illustrate survival estimates in regard of the accumulation of these factors

In multivariate Cox hazard regression analysis, a postsurgical KPS ≥ 70 (HR0.53 95% CI: 0.38‐0.71; *P* < .0001), a controlled primary disease (HR 0.67 95% CI: 0.55‐0.82; *P* < .0001), postoperative radiotherapy (HR 0.65 95% CI: 0.52‐0.82) and systemic therapy (HR 0.55 95% CI: 0.45‐0.68; *P* < .0001), as well as a number of less than four BM (HR 0.63 95% CI: 0.50‐0.80; *P* < .0001) remained independent and significant predictors of survival (Table 3). The contribution of these factors is displayed in Figure 3.

## 4 DISCUSSION

The major finding of this study was that a postsurgical improvement of the functional status in BM patients increases the likelihood of receiving further systemic treatment. In turn, systemic treatment following surgical and adjuvant radio‐therapeutic treatment of BM strongly determined the patients´ postsurgical survival.

This observation questions the traditional dogma of BM predominantly defining the patients´ prognosis: for a long time the occurrence of BM was considered a patient's end stage of disease with palliative WBRT or best supportive care being the only therapeutic options left. However, the attitude to further oncological treatment after diagnosis of BM is changing toward a comprehensive and ongoing systemic therapy, particularly since evolving pharmaceutical systemic treatment options may significantly prolong survival despite cerebral tumor spread. Novel small molecule compounds that either target cancer‐specific driver mutations or modulate the patients´ antitumor immune response were both shown to display cerebral efficacy in selected tumors.^3^, ^4^, ^5^, ^11^, ^21^

Until now, the clinical status – measured by KPS – has been an important established tool to decide whether a patient will receive tumor‐specific treatment or best supportive care.^22^ Hence, as shown in this study, a significant improvement of neurological symptoms resulting from BM resection will have a significant impact on the treatment structure and outcome in the affected patients. Therefore, BM should no longer be considered an a priori fatal development in the patients´ disease and consequently not hinder further therapy.

In this context, most previous studies reporting outcome after surgery for BM must be put into perspective, since observed differences in survival have either been attributed to BM resection only, or mostly in comparison to, or as an adjunct to radiotherapy.^12^, ^13^, ^23^ However, the impact of systemic treatment after surgery, as shown for example by Mc Hugh ^24^ – if indeed administered – has so far received little attention. Most frequently, the cause of death was also neglected. This causality is reflected by the presented data, where patients showed a clear benefit from surgery, as seen by improved NPS and KPS scores, which in turn correlated with the induction or continuation of systemic treatment. The latter correlates directly with the surgery‐related shift of patients with a poor prognosis into better RPA classes, in turn resulting in improved survival rates.

The presented cohort is representative and comparable to previous studies, even though it included patients with multiple metastases,^1^, ^14^, ^16^, ^17^, ^18^ who in clinical practice are usually not considered for surgery. In this context, the importance of the individual number of cerebral metastases should be seriously considered: regarding the entire cohort (comprising patients receiving further chemotherapy and those who did not), only multiple (≥4 BM) showed a negative impact on survival. For that reason, the traditional focus on singular or solitary metastases being candidates for resection may be revised, although it is still part of current guidelines.^25^ Noteworthy, previous studies establishing such recommendations were conducted prior to the wide‐spread availability of magnetic resonance imaging, and are therefore only based on computed tomography. Thus, the mere number of cerebral lesions may lose its traditional outstanding importance for decision making in neurosurgery.

Consequently, the role of surgery for symptomatic BM, apart from its importance in the acute situation of severe neurologic deterioration (eg by tumor‐associated hemorrhages or obstruction of cerebrospinal fluid pathways), is reasonable in the context of an interdisciplinary therapeutic approach, including systemic treatment. Particularly since in the future, further molecular analysis of BM may gain importance, changes in molecular signatures in BM compared to the primary tumors^26^, ^27^ will mean sufficient material for molecular analysis is needed. The most prominent aspect influencing the decision for or against surgery therefore must be: will metastasectomy positively change the neurological condition of the patient with a high probability, to enable further specific treatment? This question is self‐evident for patients in acute, life‐threatening conditions due to large masses in the posterior fossa, but according to our data it is also relevant for patients with rather small tumors that nevertheless cause severe symptoms due to eloquent location or severe edema. In this study, this rational is responsible for the surgical treatment of patients even with a very poor KPS, when the poor clinical status presumably resulted from neurological impairment due to the brain metastasis.

Conversely, surgery bears a considerable peri‐procedural risk, which can be frequently under‐reported.^1^ Since the complication rate was rather high compared to data reporting other cranial procedures, and the incidence of complications was a negative predictive factor for survival, the potential surgical risk must also be assessed thoroughly in the context of comorbidity. Therefore the indication for surgery has to be made carefully, fully aware that the patient´s remaining life‐time may be too limited to recover from any new neurological deficits caused by surgery, perhaps leading to exclusion from further therapy. However, surgical risks for this patient population must also be compared to other treatment modalities.

The issue of the optimal local treatment after surgery (focal radiotherapy, WBRT, radiosurgery or a combination of these) was not addressed in this study. Since the number of metastases was highly divergent, and radio‐oncological treatment‐paradigms have changed over the last decade, the applied treatment concepts resulted in a heterogeneous cohort. Moreover, evaluation of the efficacy of different radiotherapeutic measures was not within the scope of this study.

In the presented cohort, the primary entity did not significantly influence the outcome, which is explained by the heterogeneity of the tumors, which nowadays could be defined by analyzing molecular signatures. Since these patterns define the response to systemic treatment with targeted therapies, future analyses focusing on molecular tumor subtypes could help define selected cohorts that would particularly benefit from BM surgery in combination with further specific systemic treatments.

## 5 AUTHORS´ CONTRIBUTIONS

Stefan Grau and Martin Proescholdt: conceptualization, data curation, formal analysis, statistical analysis and interpretation, supervision of local contributors (Cologne and Regensburg), substantially revised the manuscript. Petra Schoedel and Stephanie Juenger: data collection, statistical calculations, original draft. H. Christian Reinhardt: methodology and data interpretation from an oncological point of view, critical review. Nils‐Ole Schmidt and Roland H. Goldbrunner: resources, review of the manuscript. All authors have approved the submitted version and have agreed to be accountable for the author's own contributions and to ensure that questions related to the accuracy of any part of the work, even ones in which the author was not personally involved, are appropriately investigated, resolved, and the resolution documented in the literature.

## CONFLICT OF INTEREST

HCR received consulting and lecture fees from Abbvie, Astra‐Zeneca, Vertex and Merck. HCR received research funding from Gilead Pharmaceuticals. The other authors (PS, SJ, NOS, MW, RHG, MP, SG) declare no conflict of interest.

## Data Availability

The data that support the findings of this study are available on request from the corresponding author. The data are not publicly available due to privacy or ethical restrictions.
